# Aldosterone Suppresses Endothelial Mitochondria through Mineralocorticoid Receptor/Mitochondrial Reactive Oxygen Species Pathway

**DOI:** 10.3390/biomedicines10051119

**Published:** 2022-05-12

**Authors:** Shih-Yuan Peng, Cheng-Hsuan Tsai, Xue-Ming Wu, Hsin-Hsiu Huang, Zheng-Wei Chen, Bo-Ching Lee, Yi-Yao Chang, Chien-Ting Pan, Vin-Cent Wu, Chia-Hung Chou, Chi-Sheng Hung, Che-Wei Liao, Yen-Hung Lin

**Affiliations:** 1 Department of Internal Medicine, Division of Cardiology, College of Medicine, National Taiwan University Hospital, National Taiwan University, Taipei 100, Taiwan; sypeng0302.12@gmail.com (S.-Y.P.); d09421005@ntu.edu.tw (C.-H.T.); hsinhsiu@gmail.com (H.-H.H.); petrehcs@gmail.com (C.-S.H.); 2 Graduate Institute of Clinical Medicine, College of Medicine, National Taiwan University, Taipei 100, Taiwan; rollerpapa@mail.chihlee.edu.tw; 3 Department of Internal Medicine, Taoyuan General Hospital, Taoyuan 330, Taiwan; ad224413@ms2.hinet.net; 4 Department of Internal Medicine, National Taiwan University Hospital, Yun-Lin 640, Taiwan; zwchen1987@ntu.edu.tw (Z.-W.C.); pan.chienting.m@gmail.com (C.-T.P.); 5 Department of Medical Imaging, College of Medicine, National Taiwan University Hospital, National Taiwan University, Taipei 100, Taiwan; bochinglee@gmail.com; 6 Division of Cardiology, Cardiovascular Medical Center, Far Eastern Memorial Hospital, New Taipei City 220, Taiwan; 7 Center of General Education, Chihlee University of Technology, New Taipei City 220, Taiwan; 8 Department of Internal Medicine, Division of Nephrology, College of Medicine, National Taiwan University Hospital, National Taiwan University, Taipei 100, Taiwan; dr.vincentwu@gmail.com; 9 Department of Obstetrics and Gynecology, College of Medicine, National Taiwan University Hospital, National Taiwan University, Taipei 100, Taiwan; ch640124@gmail.com; 10 Department of Medicine, National Taiwan University Cancer Center, Taipei 106, Taiwan; 11 Cardiovascular Center, National Taiwan University Hospital, Taipei 100, Taiwan

**Keywords:** aldosterone, primary aldosteronism, endothelial cell mitochondria, mitochondrial oxidative stress

## Abstract

Excessive aldosterone secretion causes endothelial dysfunction, vascular inflammation, and vascular fibrosis in patients with primary aldosteronism (PA). Endothelial function is closely related to endothelial mitochondria. However, the effects of elevated aldosterone levels on endothelial mitochondria remain unclear. In this study, we used primary cultured human umbilical vein endothelial cells (HUVECs) to investigate the effects of aldosterone on endothelial mitochondria. Mineralocorticoid receptor (MR) small interfering (si)RNA or glucocorticoid receptor (GR) siRNA were used to confirm the pathway by which aldosterone exerts its effects on the mitochondria of HUVECs. The results showed that excess aldosterone suppressed mitochondrial DNA copy numbers, anti-mitochondrial protein, and SOD2 protein expression in a dose- and time-dependent manner. These effects were attenuated by treatment with MR siRNA, but not with GR siRNA. Furthermore, it was attenuated by treatment with a mitochondria-targeted antioxidant (Mito-TEMPO, associated with mitochondrial reactive oxygen species (ROS) production), but not N-acetyl-L-cysteine (associated with cytosolic ROS production), which suggests that the process was through the mitochondrial ROS pathway, but not the cytosolic ROS pathway. In conclusion, aldosterone excess suppressed endothelial mitochondria through the MR/mitochondrial ROS pathway.

## 1. Introduction

Primary aldosteronism (PA) is a common and curable secondary hypertensive disorder [1]. The adrenal gland hyperplasia or adrenal adenoma in PA can lead to excessive aldosterone secretion. The excessive aldosterone in PA, which is unresponsive to renin regulation induces cardiac remodeling including fibrosis and hypertrophy and it is associated with worse cardiovascular outcomes [2]. Patients with PA have more left ventricular remodeling than patients with essential hypertension [3]. This excess aldosterone activates mineralocorticoid receptors (MRs) [4], resulting in vascular inflammation [5], increased oxidative stress [6], endothelial dysfunction [7], and vascular remodeling [8].

The vascular endothelium plays a crucial role in maintaining vascular stability and adapting the cardiovascular system to environmental changes [9]. Endothelial dysfunction is involved in many cardiovascular diseases, including hypertension, atherosclerosis, and heart failure [10,11]. Essential hallmarks of endothelial dysfunction include increased reactive oxygen species (ROS), increased pro-inflammatory factors, and insufficient nitric oxide (NO) bioavailability [12]. Excess aldosterone performs an important role in the pathogenesis of endothelial dysfunction, and contributes to vascular tone dysfunction, vascular inflammation, atherosclerosis, and vascular remodeling [13]. Aldosterone directly affects ROS generation through MR-dependent activation of NADPH oxidase in glomerular mesangial cells [14].

Mitochondrial signaling is an essential function which regulates ROS production [15]. Excessive mitochondrial ROS production is associated with cardiovascular risk [16,17], and also promotes inflammation and reduces the bioavailability of NO, resulting in endothelial dysfunction [11]. Taken together, aldosterone performs an important role in endothelial dysfunction, and mitochondria may be the orchestrator of endothelial function. Normal angiogenesis and vasodilation of endothelial cells are related to mitochondrial dynamics. In addition, mitochondria and endoplasmic reticulum cooperate to regulate calcium trafficking, thereby controlling endothelial function, including endothelial NO synthase activation, barrier function, and angiogenesis [18]. However, the effect of aldosterone on endothelial cell mitochondria and the molecular mechanisms are still unclear. Therefore, this study aimed to investigate the effect of excess aldosterone on endothelial mitochondria and to explore its mechanism in human umbilical vein endothelial cells (HUVECs).

## 2. Materials and Methods

### 2.1. Preparation of Human Umbilical Vein Endothelial Cells

HUVECs were purchased from Cell Applications (San Diego, CA, USA) and cultivated in Endothelial Cell Growth Medium (Cell Applications, San Diego, CA, USA). The HUVECs were cultivated in fibronectin (#8248, ScienCell, Carlsbad, CA, USA) coated dishes in a humidified atmosphere of 95% air and 5% CO_2_ at 37 °C. The cells were not cultivated for more than three passages.

### 2.2. Preparation of Reagents and Chemical Inhibitors

Aldosterone and N-acetyl-L-cysteine ((NAC) an antioxidant) were purchased from Sigma (St Louis, MO, USA), and Mito-TEMPO (a mitochondria-targeted antioxidant) was purchased from Enzo Life Sciences (Farmingdale, NY, USA). Aldosterone and Mito-TEMPO were dissolved in dimethyl sulfoxide (DMSO), and NAC was dissolved in double-distilled water (ddH2O).

### 2.3. Small Interfering (si)RNA and Reagents

GR siRNA (sc-35505), MR siRNA (sc-38836), and its scramble (Scr) control (sc-37007) were purchased from Santa Cruz Biotech (Santa Cruz, CA, USA). siRNA Transfection Reagent (sc-29528) (Santa Cruz, CA, USA) was used as a lipid reagent for transfection. The HUVECs were transfected with siRNA in serum-free Opti-MEM medium (Invitrogen, Carlsbad, CA, USA).

### 2.4. Mitochondrial DNA Copy Number Detection

Total DNA from the HUVECs was extracted using a QIAamp DNA Mini Kit (Qiagen, Valencia, CA, USA). The relative mitochondrial DNA copy number was detected through quantitative reverse transcription-polymerase chain reaction (qRP-PCR) using mitochondrial DNA primer: Cyt b forward 5′-TCA CCA GAC GCC TCA ACC GC-3′, reverse 5′-GCC TCG CCC GAT GTG TAG GA-3′, COII forward 5′-GGC ACA TGC AGC GCA AGT AGG-3′, and reverse 5′-GGC GGG CAG GAT AGT TCA GAC G-3′ [19]. The mitochondrial DNA was corrected with nuclear DNA β-actin gene as forward 5^′^-GCA AAG TTC CCA AGC ACA-3^′^, and reverse 5^′^-AAG CAA GCA GCG GAG CAG-3′ [20].

### 2.5. Efficacy of the MR and GR siRNA

A PCR was performed on the total RNA harvested from HUVECs, using a hMR sense primer 5′-AAATCACAC GGCGACCTGTCGT-3′; a hMR antisense primer 5′-ATGGCATCCTGAAGCCTCATCC-3′; a hGR sense primer 5′-GGAATAGGTGCCAAGGATCTGG-3′; and a hGR antisense primer 5′-GCTTACATCTGGTCTCATGCTGG-3′. The PCR consisted of a 2 min denaturing step at 95 °C; followed by 32 cycles of denaturing at 95 °C and annealing at 57 °C and extending at 72 °C; and finishing with an extension at 72 °C for 5 min. The specificity of PCR products was confirmed by agarose gel electrophoresis.

### 2.6. Mitochondria Immunofluorescence Staining

A coverslip was placed on a six-well culture dish which was coated with Bovine Plasma Fibronectin (#8248, ScienCell, Carlsbad, CA, USA), and the HUVECs were seeded. After fixation, the cells were incubated with primary anti-mitochondrial antibodies (ab92824, Abcam, Cambridge, UK), followed by incubation with fluorescence-conjugated secondary antibodies. The slides were mounted, and the next day they were photographed under a fluorescence microscope.

### 2.7. Western Blot Analysis

Cell lysate was mixed with 5-fold diluted Bio-Rad Protein Assay Dye Reagent Concentrate (Bio-Rad, Hercules, CA, USA) to measure the absorbance value. The cell lysate (20 μg) was used for the Western blot analysis with 3-5 mL of primary antibodies: glyceraldehyde-3-phosphate dehydrogenase (GAPDH) (MAB374, EMD Millipore, Billerica, MA, USA), anti-mitochondria antibody (ab92824), and SOD2 (ab13533). Signals were detected by adding Western Lightning Plus (PerkinElmer, Waltham, MA, USA). A digital imaging system (Bio Pioneer Tech Co., New Taipei City, Taiwan) was used to detect the signals, which were further analyzed using ImageJ^®^ software.

### 2.8. Mitochondria ROS (mtROS) Detection

The mtROS formation in the HUVECs was determined using MitoSOX™ (M36008, Invitrogen™, Carlsbad, CA, USA). After experimental treatment, the cells were washed with PBS, and 5 uM of MitoSOX solution was added. The cells were then incubated for 15 min at 37 °C and protected from light, followed by incubation with 100 nM of MitoTRacker (M7514, Invitrogen™, Carlsbad, CA, USA) working solution for 30 min at 37 °C and protected from light. The slides were mounted and photographed under a fluorescence microscope.

### 2.9. Detection of Reactive Oxygen Species

ROS formation in the HUVECs was measured using the fluorescent dye 2′,7′-dichlorofluorescein diacetate (DCF-DA, Sigma, St Louis, MO, USA). First, the HUVECs were seeded onto fibronectin-coded slides and placed in six-well plates. After incubation under experimental conditions, the cells were incubated with DCF-DA (5 mM) for 30 min at 37 °C, and ROS accumulation was measured using fluorescence microscopy. DCF-DA fluorescence emission was measured at 530 nm with excitation at 488 nm using a Beckman Coulter DTX 880 multimode detector (Beckman Coulter, Brea, CA, USA).

### 2.10. Statistical Analysis

Data are shown as mean ± standard deviation. Each cell experiment was performed in triplicate. Differences between variants were compared using a *t*-test for two independent groups. The significance level was set at *p* < 0.05. A statistical analysis was performed using SPSS version 25 for Windows (SPSS Inc., Chicago, IL, USA).

## 3. Results

### 3.1. Aldosterone Treatment Reduced HUVEC Mitochondrial DNA

**Figure 1:**
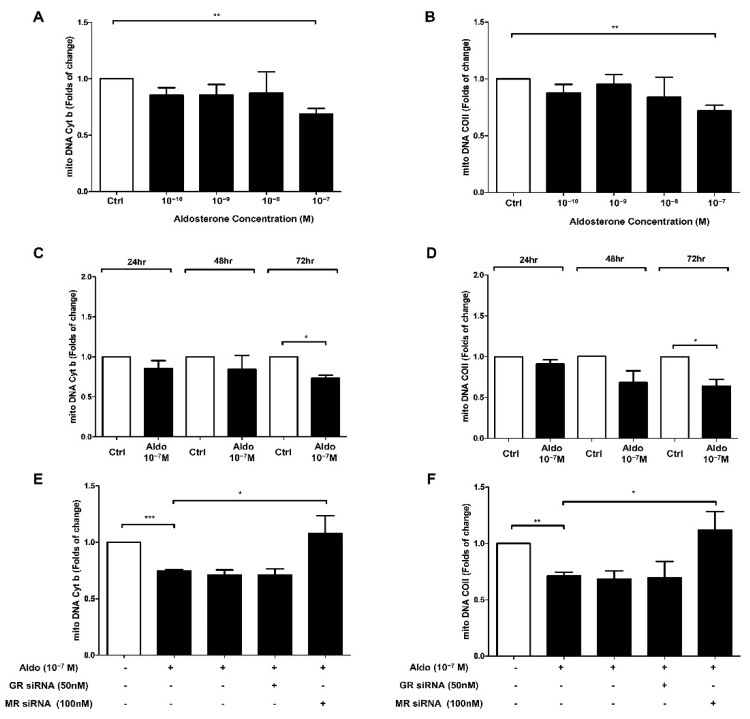
Aldosterone inhibits mitochondrial DNA in a dose-dependent and time-dependent manner through the MR pathway in HUVECs. (**A**) The mitochondrial DNA Cyt b copy number and (**B**) the mitochondrial DNA COII copy number were quantified by qPCR in HUVECs treated with different aldosterone concentrations (10^−10^, 10^−9^, 10^−8^, 10^−7^ M) for 72 h. (**C**) The mitochondrial DNA Cyt b copy number and (**D**) the mitochondrial DNA COII copy number were quantified by qPCR at 24, 48, and 72 h in HUVECs treated with 10^−7^ M aldosterone. (**E**) The mitochondrial DNA Cyt b copy number and (**F**) the mitochondrial DNA COII copy number were quantified by qPCR in HUVECs at 72 h after transfection of the HUVECs with GR siRNA (50 nM) or MR siRNA (100 nM) for 6 h before 10^−7^ M aldosterone treatment. * *p* < 0.05, ** *p* < 0.01, *** *p* < 0.001, compared between two groups using the *t*-test.

HUVECs were treated with different concentrations of aldosterone (10^−10^, 10^−9^, 10^−8^ and 10^−7^ M) for 72 h. The mitochondrial DNA copy number (both mitochondrial DNA Cyt b and COII) significantly decreased after 72 h of 10^−7^ M aldosterone treatment (Figure 1A,B). We then examined the timing (24, 48, and 72 h) of the decrease in HUVEC mitochondrial DNA copy number with 10^−7^ M aldosterone. We found that both mitochondria DNA Cyt b and COII significantly decreased after 72 h of treatment (Figure 1C,D).

### 3.2. Aldosterone Suppressed HUVEC Mitochondrial DNA via MR Activation

We then used MR siRNA and GR siRNA to evaluate the mechanisms underlying the aldosterone-induced mitochondrial DNA suppression in HUVECs. First, we examined the expression of GR mRNA and MR mRNA in HUVEC cells. Compared with control siRNA, the MR and GR siRNA could suppress the MR and GR gene expression, respectively. The efficacy of GR siRNA and MR siRNA are shown in Supplementary Figure S1.

After 6 h of siRNA treatment and then 72 h of 10^−7^ M aldosterone treatment, MR siRNA significantly inhibited the effects of aldosterone on reducing the mitochondrial DNA copy number, but this effect was not seen with GR siRNA (Figure 1E,F). We also used an MR antagonist (eplerenone) and a GR antagonist (RU486) to evaluate the mechanisms of aldosterone-induced mitochondrial DNA inhibition in HUVECs. The results showed that eplerenone significantly inhibited the effects of aldosterone on reducing the mitochondrial DNA copy number (Supplementary Figure S2).

### 3.3. Aldosterone Treatment Decreased Mitochondria-Specific Protein in HUVECs

**Figure 2:**
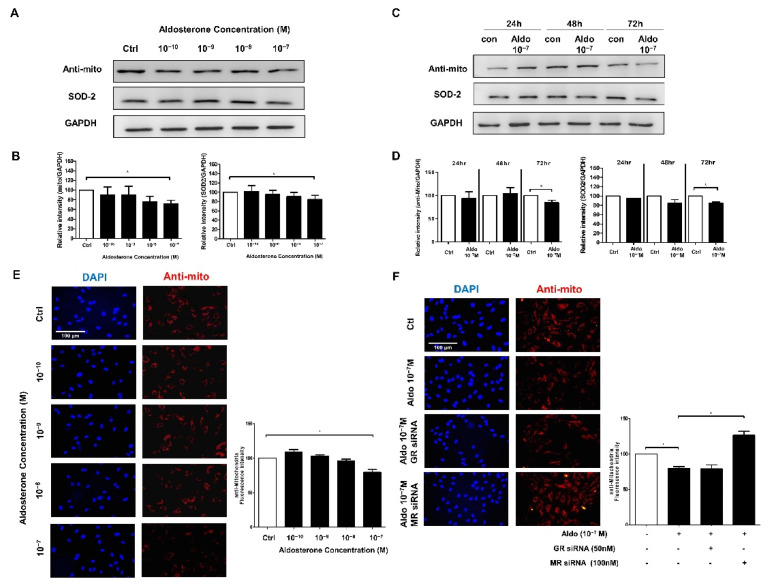
Aldosterone inhibits mitochondrial and SOD2 protein expression in a dose-dependent and time-dependent manner through the MR pathway in HUVECs. (**A**) Western blot analysis of mitochondrial and SOD2 protein in HUVECs treated with different aldosterone concentrations (10^−10^, 10^−9^, 10^−8^, 10^−7^ M) for 72 h. (**B**) Relative mitochondrial and SOD2 protein ratios are indicated in GAPDH using Image J software in HUVECs treated with different aldosterone concentrations (10^−10^, 10^−9^, 10^−8^, 10^−7^ M) for 72 h. (**C**) Western blot analysis of the mitochondrial and SOD2 protein in HUVECs treated with 10^−7^ M aldosterone for 24, 48, and 72 h. (**D**) Relative mitochondrial and SOD2 protein ratios are indicated in GAPDH using Image J software in HUVECs treated with 10^−7^ M aldosterone for 24, 48, and 72 h. (**E**) HUVECs were treated with different concentrations of aldosterone (10^−10^, 10^−9^, 10^−8^, 10^−7^ M) and vehicle (equal volume of DMSO) for 72 h. Mitochondria were stained red with anti-mitochondrial antibodies, and cell nuclei were stained blue with DAPI. Quantitative fluorescence intensity was analyzed using Image J software. (**F**) HUVECs were transfected with GR siRNA (50 nM) or MR siRNA (100 nM) for 6 h before 10^−7^ M aldosterone treatment. After 72 h, mitochondria were stained red with anti-mitochondrial antibodies, and cell nuclei were stained blue with DAPI. Representative images were captured using a fluorescence microscope with 40x magnification. Scale bar: 100 μm. Quantitative fluorescence intensity was analyzed using Image J software. * *p* < 0.05, compared between two groups using the *t*-test.

To evaluate the expression of the mitochondria-specific antioxidant enzyme SOD2 and the amount of mitochondria, HUVECs were treated with different concentrations of aldosterone (10^−10^, 10^−9^, 10^−8^, and 10^−7^ M) for 72 h. The expressions of SOD2 and mitochondrial protein were significantly decreased after 72 h of treatment (Figure 2A,B). We then used 10^−7^ M aldosterone to examine the timing (24, 48, and 72 h) of the decrease in mitochondria-specific proteins in HUVECs. The SOD2 and mitochondria-specific protein expressions were significantly decreased at 72 h (Figure 2C,D).

HUVECs were then treated with different aldosterone concentrations (10^−10^, 10^−9^, 10^−8^, and 10^−7^ M) for 72 h, and anti-mitochondrial antibodies were used to detect the amount of mitochondria after treatment. The fluorescence intensity of HUVEC mitochondria was significantly decreased after 10^−7^ M aldosterone treatment for 72 h (Figure 2E).

### 3.4. Aldosterone Suppressed HUVEC Mitochondria-Specific Protein via MR Activation

We then stained the mitochondria treated with siRNA and 10^−7^ M aldosterone for 72 h in HUVECs. MR siRNA significantly inhibited the reduction in fluorescence intensity of HUVEC mitochondria by 10^−7^ M aldosterone, but this effect was not seen with GR siRNA (Figure 2F).

### 3.5. Aldosterone-Induced Mitochondrial ROS in HUVECs

**Figure 3:**
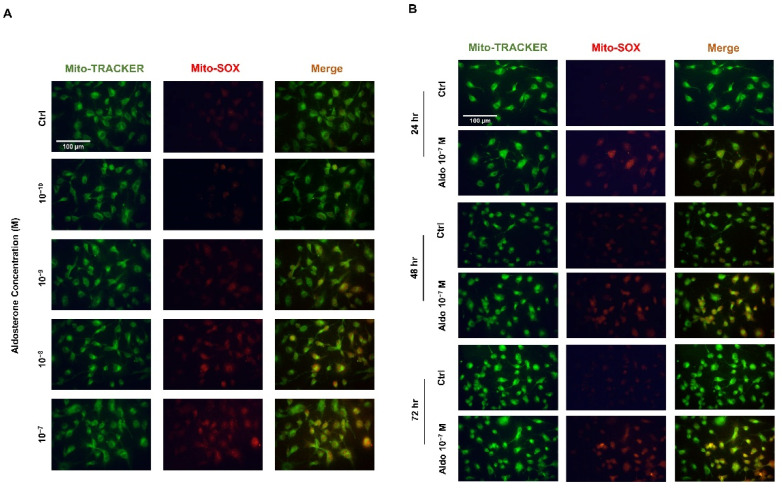
Aldosterone induces the production of mitochondrial ROS in a time-dependent and dose-dependent manner in HUVECs. (**A**) HUVECs were treated with different aldosterone concentrations (10^−10^, 10^−9^, 10^−8^, 10^−7^ M) for 72 h. (**B**) HUVECs were treated with 10^−7^ M aldosterone for 24, 48, and 72 h. MtROS was stained red with MitoSox, and mitochondria were stained green with MitoTracker. The representative images were captured using a fluorescence microscope with 40x magnification. Scale bar: 100 μm.

HUVECs were treated with different concentrations of aldosterone (10^−10^, 10^−9^, 10^−8^, and 10^−7^ M) for 72 h, and the results showed that mtROS increased with the dose of aldosterone (Figure 3A). HUVECs were then treated with 10^−7^ M aldosterone for 24 h, 48 h, and 72 h, and the results showed that mtROS increased in time-dependent manner (Figure 3B).

### 3.6. Mitochondria-Targeted Antioxidant Treatment Significantly Decreased mtROS and Restored Mitochondrial Function

**Figure 4:**
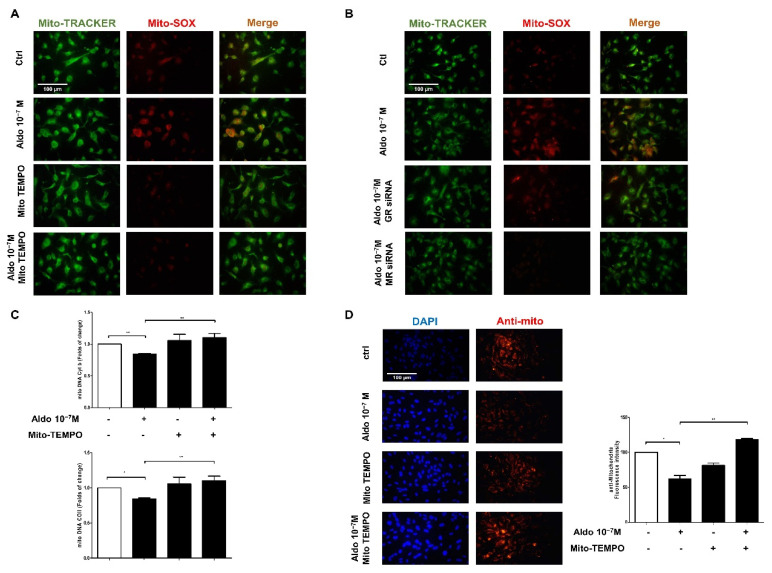
Mito-TEMPO reduces the function of mitochondrial in the aldosterone-stimulated HUVECs. HUVECs were treated with Mito-TEMPO (10 uM) for 2 h before being treated with 10^−7^ M aldosterone. (**A**) After 24 h, immunofluorescence images showed mtROS production. (**B**) HUVECs were transfected with GR siRNA (50 nM) or MR siRNA (100 nM) for 6 h and then treated with 10^−7^ M aldosterone. MtROS was stained red with MitoSox, and mitochondria were stained green with MitoTracker. (**C**) The mitochondrial DNA Cyt b copy number (up) and mitochondrial DNA COII copy number (down) were quantified by qPCR. (**D**) Mitochondria were stained red with anti-mitochondrial antibodies, and cell nuclei were stained blue with DAPI. Representative images were captured using a fluorescence microscope with 40x magnification. Scale bar: 100 μm. Quantitative fluorescence intensity was analyzed using Image J software. * *p* < 0.05, ** *p* < 0.01, compared between two groups using the *t*-test.

The mitochondria-targeted antioxidant, Mito-TEMPO, was used to examine the mechanisms of aldosterone-induced mtROS in HUVECs. HUVECs were pretreated with Mito-TEMPO for 2 h, and then treated with 10^−7^ M aldosterone. The results showed that Mito-TEMPO reduced 10^−7^ M aldosterone-induced mtROS production (Figure 4A). MR siRNA also decreased 10^−7^ M aldosterone-induced mtROS production (Figure 4B). In addition, Mito-TEMPO significantly restored the mitochondrial DNA copy number in HUVECs treated with 10^−7^ M aldosterone (Figure 4C). Mito-TEMPO also significantly restored the decreased fluorescence intensity of HUVEC mitochondria treated with 10^−7^ M aldosterone (Figure 4D). Aldosterone-induced mtROS decreased mitochondrial DNA copy number and mitochondrial-specific protein via the MR pathway in HUVECs; this effect can be rescued by the mitochondria-targeted antioxidant Mito-TEMPO.

### 3.7. NAC Could Not Restore Mitochondrial Function

**Figure 5:**
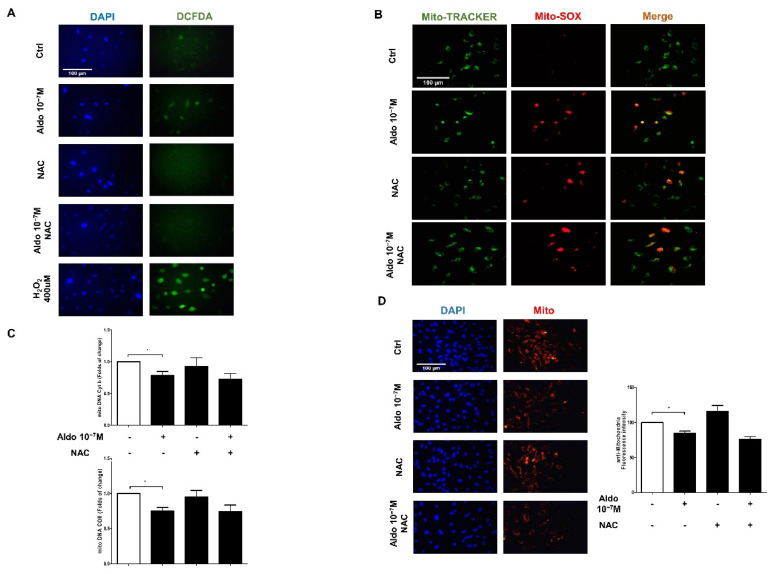
NAC cannot rescue the mitochondrial function reduction in the aldosterone-stimulated HUVECs. HUVECs were treated with NAC (100 uM) for 1 h before being treated with 10^−7^ M aldosterone. (**A**) Cellular ROS in HUVECs were shown using immunofluorescence imaging with a DCFDA probe after treatment with NAC (100 uM) for 1 h and then 10^−7^ M aldosterone. ROS were stained green with the DCFDA probe, and cell nuclei were stained blue with DAPI. (**B**) Immunofluorescence images showed mtROS production. mtROS was stained red with MitoSox, and mitochondria were stained green with MitoTracker. (**C**) The mitochondrial DNA Cyt b copy number (up) and mitochondrial DNA COII copy number (down) were quantified by qPCR. (**D**) Mitochondria were stained with anti-mitochondrial antibodies, and cell nuclei were stained blue with DAPI. Representative images were captured using a fluorescence microscope with 40x magnification. Scale bar: 100 μm. Quantitative fluorescence intensity was analyzed using Image J software. * *p* < 0.05, compared between two groups using the *t*-test.

NAC is an antioxidant which is commonly used to identify and inhibit ROS production. We used NAC to evaluate the effects of aldosterone-induced cellular ROS on endothelial mitochondria. HUVECs were pre-treated with NAC for 1 h and then treated with aldosterone for 4 h. The fluorescence intensity of ROS increased after aldosterone treatment, and NAC decreased the fluorescence intensity of ROS (Figure 5A). However, NAC could not reduce aldosterone-induced mtROS production (Figure 5B). In addition, NAC could not rescue the reduction in aldosterone-induced mitochondrial DNA copy number (Figure 5C) or the fluorescence intensity of mitochondria (Figure 5D).

**Figure 6:**
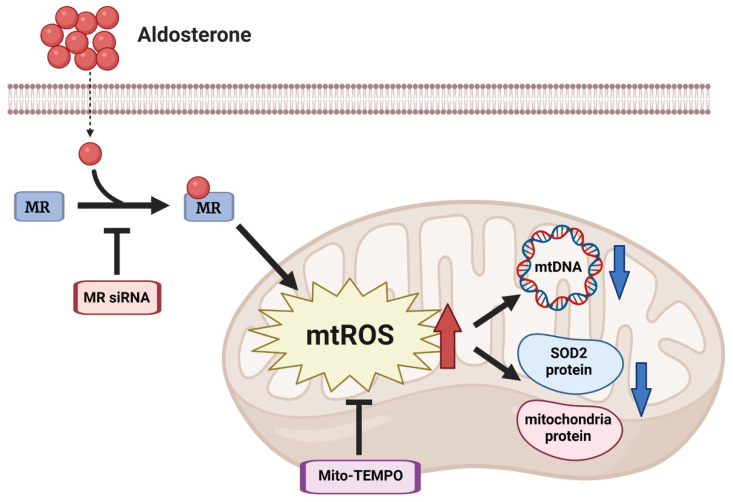
Schematic of the signaling of aldosterone suppresses endothelial mitochondria in HUVECs. Aldosterone suppresses endothelial mitochondrial DNA and mitochondria-specific proteins through the MR/mitochondrial ROS (mtROS) pathway.

The possible signaling pathway of aldosterone-induced HUVEC mitochondrial suppression is summarized in Figure 6.

## 4. Discussion

In this study, excess aldosterone led to the suppression of endothelial mitochondria in HUVECs through the MR/mtROS pathway. In addition, mitochondrial DNA copy number and mitochondria-specific proteins were suppressed in HUVECs after aldosterone treatment.

Patients with PA have excessive endogenous secretion of aldosterone which is unresponsive to renin regulation [2]. Clinical subtypes of PA include unilateral aldosterone-producing adenoma, unilateral adrenal hyperplasia, and bilateral adrenal hyperplasia. Adrenalectomy is the standard treatment for the unilateral PA and medical therapy for bilateral PA [21,22]. These treatment options are aimed to decrease the detrimental effects of excess aldosterone. The known effects of aldosterone are mainly through regulating MRs [23]. MRs are expressed in cardiomyocytes, endothelial cells, vascular smooth muscle cells, adipocytes, and hypothalamic neurons [24]. Aldosterone promotes vascular remodeling by inducing vascular smooth muscle cell proliferation and fibrosis after endothelial injury in MR knock-out (MR-KO) mice [25]. This supports the critical role of aldosterone in the vasculature.

Blood vessels are composed of connective tissue, fibroblasts, endothelial cells, and vascular smooth muscle cells. The endothelium plays an essential role in regulating cardiovascular function, including vascular tone, vasculature, and cellular activity. Activated endothelial cells can release various cytokines, chemokines, and growth factors to promote endothelial cell proliferation, migration, and infiltration [26].

The vascular endothelium plays an important role in maintaining vascular stability and adapting the cardiovascular system to environmental changes [27]. Vascular inflammation caused by endothelial cells with an inflammatory phenotype leads to endothelial dysfunction and the progression of cardiovascular disease [11]. In our previous study, we found that patients with PA had problems with blood flow-mediated dilation, and that the blood flow-mediated dilation was improved after surgery, indicating that aldosterone affects endothelial function [28]. Another study showed that interleukin-6 was elevated in the blood of patients with PA, and related studies have suggested that interleukin-6 may be secreted by vascular endothelial cells and causes myocardial fibrosis [29].

Several explanatory mechanisms for the association between excess aldosterone and endothelial dysfunction have been postulated. In endothelial cells, aldosterone activates NADPH oxidase through the MR pathway to induce superoxide production. In addition, aldosterone promotes ICAM-1 transcription and leukocyte adhesion in coronary endothelial cells [30]. Glucose-6-phosphate dehydrogenase (G6PD) has also been shown to regulate endothelial function, and aldosterone inhibits the expression of G6PD in bovine aortic endothelial cells and human coronary artery endothelial cells, thereby increasing oxidative stress and decreasing NO bioavailability [31]. In this study, we found another novel mechanism of aldosterone-induced endothelial dysfunction. In HUVECs, aldosterone significantly suppressed mitochondrial DNA and mitochondria-specific proteins in a dose- and time-dependent manner through MR activation and mtROS generation.

Mitochondrial dysfunction or mitochondrial DNA mutations are associated with cardiovascular disease [32]. Compensatory increases in the nuclear OXPHOS gene have been shown to lead to mitochondrial DNA damage with age, and to be associated with atherosclerotic occlusion of the coronary arteries [33]. Mitochondrial DNA damage has also been shown to lead to increased ROS production and atherosclerosis [34]. The accumulation of mitochondrial DNA point mutations and decreased mitochondrial DNA content with age may lead to organ failure. In addition, a reduction in mitochondrial DNA has also been associated with mitochondrial dysfunction [18].

Human mitochondrial DNA is a double-stranded circular molecule of 16569 bp. Although mitochondrial DNA encodes only 13 of all mitochondrial proteins, mitochondrial DNA damage is more susceptible to ROS than nuclear DNA. First, it lacks many of the mechanisms involved in nuclear DNA repair. Second, it is close to the electron transport chain, which is the main source of ROS production [35]. In our previous study, we found that the number of mitochondria in peripheral blood leukocytes in patients with PA was significantly lower than in patients with essential hypertension, and that it was associated with lower the myocardial mass index [36]. In addition, the ventricular tissue of aldosterone-infused C57BL/6 mice had a pathological pattern of cardiac hypertrophy that was not seen in the control group and the aldosterone also down-regulated mitochondria in the cardiomyocytes of the mice [36]. These effects of aldosterone on mitochondrial number and function were through the NOX-2/ROS system in mouse cardiomyocytes [36]. Interestingly, aldosterone did not affect endothelial cell mitochondria through the cytosolic ROS pathway but through the mitochondria pathway in the current study.

Compared with cardiomyocytes, endothelial cells contain relatively few mitochondria. For example, in rats, mitochondria form 2–6% of the cytoplasmic volume in endothelial cells and 32% in cardiomyocytes. Brain endothelial cells have higher mitochondrial content (8–11%) [18]. Therefore, the function of endothelial mitochondria is often overlooked. However, in this study, we found that the detrimental effects of aldosterone on endothelial mitochondria were through the aldosterone-MR-mtROS pathway in HUVECs. Aldosterone induced the production of mtROS in a dose- and time-dependent manner, and the mitochondria-targeted antioxidant Mito-TEMPO can rescue the effects. We also found that Mito-TEMPO can rescue the aldosterone-induced damage to endothelial mitochondrial DNA and proteins. Interestingly, the aldosterone-induced mitochondrial suppression could be attenuated by treatment with the mitochondria-targeted antioxidant associated with mtROS production (Mito-TEMPO), but not that associated with cytosolic ROS production (NAC). This indicates that the process was through the mtROS, but not cytosolic ROS pathway.

The current study has several limitations. First, the main findings of our study were from cell experiments, and it is unclear whether similar findings would be found in animal experiments and clinical conditions. However, the present study provides good evidence to support the underlying mechanisms of aldosterone-induced mitochondrial dysfunction in endothelial cells. Second, we found that aldosterone inhibited mitochondrial DNA and mitochondrial protein expression in endothelial cells; however, we did not explore the effect on mitochondrial energy metabolism in detail in this study.

## 5. Conclusions

In conclusion, we demonstrated that aldosterone excess suppressed endothelial mitochondrial DNA and mitochondria-specific proteins through the MR/mtROS pathway in HUVECs.

## Data Availability

The original contributions presented in the study are included in the article. Further inquiries can be directed to the corresponding authors.

## Supplementary Materials

The following supporting information can be downloaded at: https://www.mdpi.com/article/10.3390/biomedicines10051119/s1, Figure S1: The GR and MR gene expression following silencing with GR siRNA or MR siRNA in HUVECs.; Figure S2: Aldosterone suppressed HUVEC mitochondrial DNA via MR activation in HUVECs.
