# Supplementary material for: Aldosterone Suppresses Endothelial Mitochondria through Mineralocorticoid Receptor/Mitochondrial Reactive Oxygen Species Pathway
Source: Biomedicines. 2022 May 12;10(5):1119. doi: 10.3390/biomedicines10051119 (PMC9138689; doi:10.3390/biomedicines10051119)
Supplement: Supplementary file 1 [file biomedicines-10-01119-s001.zip › biomedicines-1658726-supplementary.pdf]

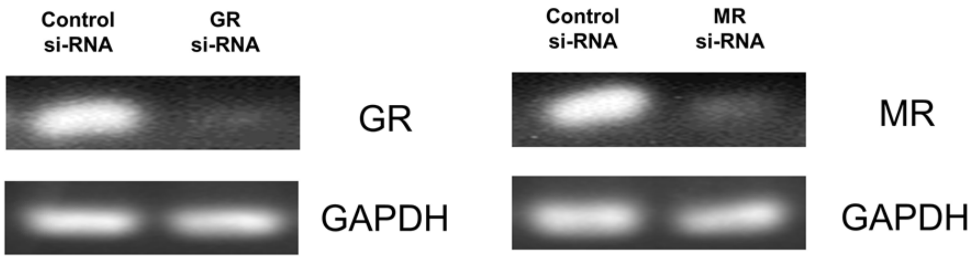

Figure S1. The GR and MR gene expression following silencing with GR siRNA or MR siRNA in HUVECs. Representative original bands of GR or MR (upper bands) and control GAPDH (lower bands) cDNA expression in HUVEVCs after 6 hours of silencing with 50nM GR siRNA or with 100 nM MR siRNA.

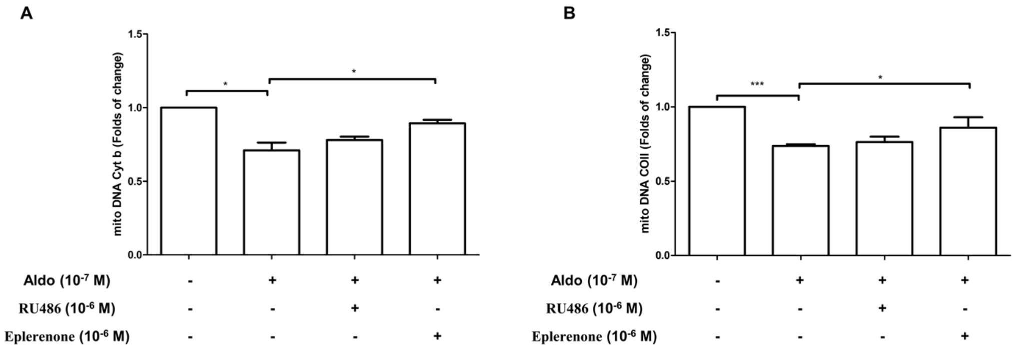

Figure S2. Aldosterone suppressed HUVEC mitochondrial DNA via MR activation in HUVECs. The mitochondrial DNA Cyt b copy number (A) and the mitochondrial DNA COII copy number (B) were quantified by qPCR in HUVECs pretreated with the MR antagonist (eplerenone) 10^{-6} M or GR antagonist (RU486) 10^{-6} M for 1 hour and 10^{-7} M aldosterone treatment for 72 hours. *$P < 0.05$, ***$P < 0.001$, compared between two groups using the t-test.
